# Intraoperative Fluid Excess Is a Risk Factor for Pancreatic Fistula after Partial Pancreaticoduodenectomy

**DOI:** 10.1155/2016/1601340

**Published:** 2016-09-21

**Authors:** Helge Bruns, Veronika Kortendieck, Hans-Rudolf Raab, Dalibor Antolovic

**Affiliations:** Department of General and Visceral Surgery, Carl von Ossietzky University of Oldenburg, Oldenburg, Germany

## Abstract

*Background*. After pancreaticoduodenectomy (PD), pancreatic fistulas (PF) are a frequent complication. Infusions may compromise anastomotic integrity. This retrospective analysis evaluated associations between intraoperative fluid excess and PF.* Methods*. Data on perioperative parameters including age, sex, laboratory findings, histology, infusions, surgery time, and occurrence of grade B/C PF was collected from all PD with pancreaticojejunostomy (PJ) performed in our department from 12/2011 till 02/2015. The glomerular filtration rate (GFR), infusion rate, and the ratio of both and its association with PF were calculated. ROC analysis was employed to identify a threshold.* Results*. Complete datasets were available for 83 of 86 consecutive cases. Median age was 66 years (34–84; 60% male), GFR was 93 mL/min (IQR 78–113), and surgery time was 259 min (IQR 217–307). Intraoperatively, 13.6 mL/min (7–31) was infused. In total, *n* = 18 (21%) PF occurred. When the infusion : GFR ratio exceeded 0.15, PF increased from 11% to 34% (*p* = 0.0157). No significant association was detected for any of the other parameters.* Conclusions*. This analysis demonstrates for the first time an association between intraoperative fluid excess and PF after PD with PJ even in patients with normal renal function. A carefully patient-adopted fluid management with due regard to renal function may help to prevent postoperative PF.

## 1. Introduction

In high volume centers, partial pancreaticoduodenectomy (PD) can be performed with acceptable morbidity and mortality [1]. While the outcome is clearly associated with surgeon and center experience, the rate of pancreatic fistulas seems not to drop below a certain level [2, 3]. Even after thousands of PD, highly experienced surgeons in high-volume centers report an almost constant or even increasing rate of pancreatic fistulas [2]. Depending on the definition, the published rate of postoperative fistulas after PD is estimated to be 20–30% [4]. Numerous interventions and techniques have been introduced and a number of standardized anastomotic techniques exist, but there is little evidence for superiority of one anastomotic technique over the other [5–13]. Isolation of the pancreaticojejunostomy (PJ) using dual-loop reconstruction has been discussed as possible intervention to decrease the rate of pancreatic fistulas but seems not to be superior to single-loop reconstruction [1].

In multivariate analyses, some risk factors for anastomotic leakage have already been identified [14–18]. Soft pancreatic texture, a history of weight loss, intraoperative blood loss, diameter of the pancreatic duct, and decreased preoperative albumin seem to be associated with leakage. Renal insufficiency has been shown to be associated with increased complication rates after pancreatic resection [19].

In general, intraoperative fluid management aims at stabilizing the patient. For some types of abdominal surgery, a restrictive fluid regimen is considered common sense: as far as liver resection is concerned, fluid management at a low central venous pressure below 5 cm H_2_O is made use of to decrease intraoperative blood loss [20–23]. Anesthesiological guidelines may vary between institutions but usually consider hemodynamic parameters, blood values, blood loss, and duration of surgery to be triggers that guide the intraoperative regimen [24]. Interstitial fluid overload due to infusion of large amounts of fluid can lead to visible edema and might compromise the anastomotic integrity [25]. For rectal resections, an increased risk of anastomotic leakage after excessive perioperative infusions has been shown [26]. In pancreatic surgery, there is evidence pointing out that the same mechanisms might be relevant. In a study focusing on normovolemic hemodilution to decrease blood loss in pancreatic surgery, no effect on blood loss was identified but an increased rate of 21.5% versus 7.7% anastomotic complications was apparent after 6250 mL versus 3900 mL of intraoperative intravenous fluid [27]. As far as postoperative fluid management is concerned, a restrictive management aiming at a fluid balance of less than 1 liter on postoperative day has been shown to be associated with decreased complication rates [28].

This study was designed to identify associations between renal function, intraoperative infusions, and occurrence of clinical relevant postoperative pancreatic fistulas after PD with PJ.

## 2. Methods

### 2.1. Patient Data

Data extraction, handling, and analysis were performed in accordance with national and institutional guidelines. All PDs with PJ performed from 12/2011 till 02/2015 were analyzed and data on patient age, indication to surgery, concomitant diseases, preoperative laboratory values, duration of surgery, blood loss, intraoperative infusions, postoperative infusions during the first 72 hours after surgery, occurrence of pancreatic fistulas, and interventions (both surgical or other) was extracted from patient folders. Glomerular filtration rate (GFR) was calculated as published elsewhere [29]. Patients with pancreaticogastrostomies were excluded. PF were considered to be clinically relevant when drainages had to be kept longer than 72 h and pancreatic enzyme levels in the drainage fluid were at least three times higher than in the serum, drainages had to be reintroduced (e.g., guided using computer tomography or ultrasound), and pancreatic enzymes were detected in the drainage fluid or patients had to undergo reoperations for complications caused by PF.

While duct diameter and pancreatic texture have been discussed as factors associated with postoperative fistula, these parameters lack clear definitions: “duct wideness” and “tissue softness” strongly depend on the individual surgeon's definition and experience. Without clear definition of both parameters prior to documentation, including this type of data in a retrospective analysis cannot be considered to be reliable. Thus, we did not include this in our analysis and have assumed an equal distribution of both parameters between groups.

### 2.2. Statistical Analysis

For descriptive statistics, median and interquartile range (IQR) or range are used unless stated otherwise. To determine a meaningful cutoff for the infusion rate : GFR ratio, receiver operating characteristics (ROC) were employed, and the area under the ROC curve (AUC-ROC) and Youden's *J* were calculated. Testing for statistical significance was performed using ANOVA and Fisher's Exact Test as appropriate. *p* values were two tailed and a value of *p* < 0.05 was considered statistically significant.

### 2.3. Surgical Technique and Postoperative Management

In our institution, PD is performed in a highly standardized fashion. Usually, single-loop reconstruction with a Warren-Cattell end-to-side PJ using poly-p-dioxanone (PDS 5.0, Johnson & Johnson Medical GmbH, Ethicon, Norderstedt, Germany) is performed. A pancreaticogastrostomy may be performed depending on the surgeon's choice. A surgical drain is routinely placed to the pancreatic anastomosis. Intraoperative and postoperative fluid management and postoperative course are routinely monitored and logged. Patients are extubated in the operating room and transferred to the intensive care unit where mobilization is started and oral fluids are introduced on the day of surgery. Solid foods are introduced depending on enteral passage. As soon as patients have been stable for at least 24 hours, they are transferred to the intermediate care ward. Drains are removed as soon as the fluid is less than 500 mL and is serous. If there is suspicion of a PF, drains are kept in place and enzyme levels are monitored in the drainage fluid.

## 3. Results

**Figure 1:**
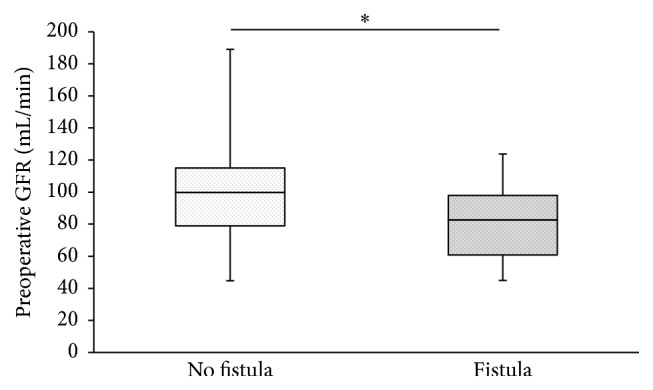
Glomerular filtration rate (GFR). In patients with postoperative pancreatic fistulas, the median GFR was 83 mL/min versus 100 mL/min in patients without fistulas (^*∗*^ *p* = 0.0219).

**Figure 2:**
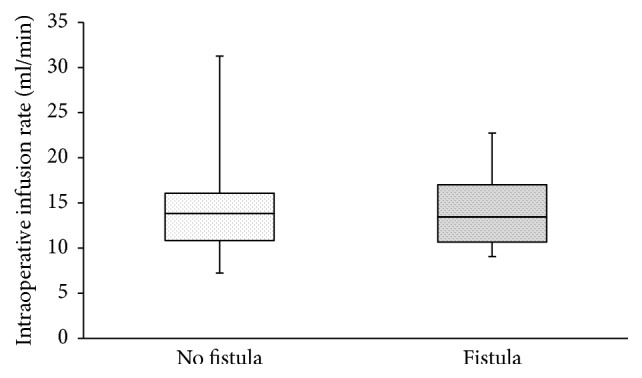
Intraoperative infusion rates (mL/min). No significant difference was detected for intraoperative infusions for patients with versus without postoperative pancreatic fistulas (*p* = 0.9633).

**Figure 3:**
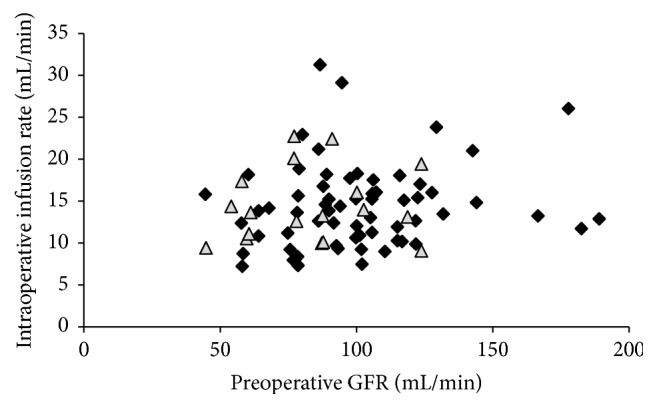
Scatterplot illustrating correlation between GFR and intraoperative infusion rate. Intraoperative infusion rates were not related to preoperative GFR (coefficient of determination: *r* ^2^ = 0.0271). Black squares: patients without fistulas. Grey triangles: patients with fistulas.

**Figure 4:**
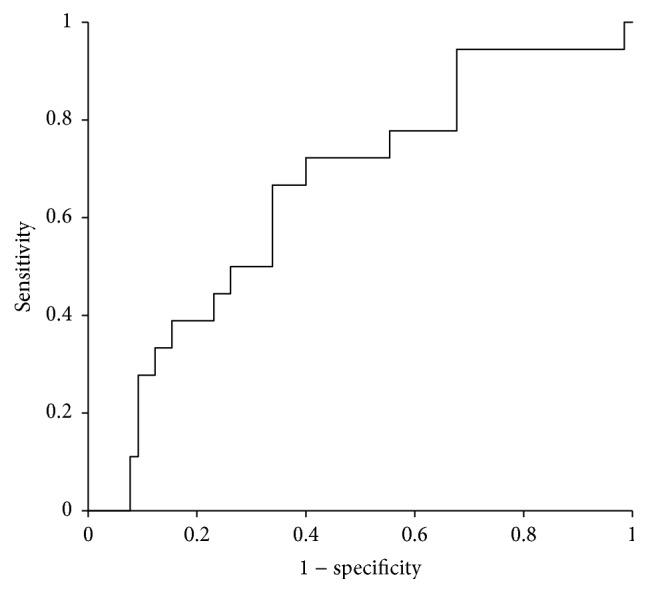
Receiver operating characteristics (ROC) curve for the infusion rate : GFR ratio. A threshold of 0.15 was detected using ROC analysis (ROC-AUC: 0.6564; Youden's *J* for 0.15: 0.3282).

In total, 83 complete datasets were available from 86 consecutive PD with PJ. In total, *n* = 18 (21%) relevant pancreatic fistulas occurred (Table 1). Median age was 66 years (range 34–84 years; 60% male), GFR was 93 mL/min (IQR 78–113 mL/min; Figure 1), and surgery time was 259 min (IQR 217–307; Table 1). Intraoperatively, 13.6 mL/min (range 7–31 mL/min) was infused (Figure 2; Table 1). Crystalloids were used in all patients during surgery and during the first 72 h after surgery. Colloids were infused in 46% of patients during surgery (56% and 43% in patients with and without fistulas; *p* = 0.4264), while 24% of patients received colloids during the first 72 h after surgery (0% versus 31% in patients with and without fistulas; *p* = 0.0046; Table 1). The amount of postoperative infusions had no effect on occurrence of pancreatic fistulas (Table 1). Except for creatinine, GFR, and postoperative infusion of colloids, no statistical differences were identified between patients with or without pancreatic fistulas (Table 1). There was no correlation between the amount of intraoperatively infused volume and GFR (Figure 3). ROC analysis identified an infusion rate : GFR ratio of 0.15 as threshold for occurrence of pancreatic fistulas (Figure 4). A significant increase of pancreatic fistulas from 11% to 34% was detected for patients below and above the identified threshold (*p* = 0.0157; Table 2).

## 4. Discussion

Perioperative fluid management strongly depends on teamwork involving both anesthesiologists and surgeons [30]. This can be quite demanding and needs experience on the anesthesiologists' side similar to surgical experience needed when complex surgery is performed. Without a balanced and careful fluid regimen, the surgery's success is jeopardized. It is a common misconception that fluid management has little effect on surgical complication rates, although this has been demonstrated frequently [22, 23, 26–28, 31]. In abdominal surgery, restrictive fluid management has repeatedly proven to be superior over dilutive regimens. In liver resection, restrictive fluid management is used to achieve a low central venous pressure and leads to decreased intraoperative blood loss, which is known to be associated with increased morbidity [22, 23]. In rectum resection, anastomotic complications increase after perioperative fluid excess [26]. In pancreatic surgery, both intraoperative and postoperative amounts of infusions have been linked to anastomotic complications [27, 28]. Excessive (and in this context: mindless) infusion management can be linked to increased postoperative complications including impaired wound healing, pulmonary complications, and intestinal paralysis [31–35]. It seems obvious that successful fluid management in pancreatic surgery needs to be balanced: while excretory renal function can visually be monitored during surgery and hemodynamic parameters are constantly made use of as triggers for infusions and pharmacological interventions, renal function is usually only considered important when already compromised. Most of our patients had a normal renal function with a GFR within normal values (Table 1, Figure 1). Nonetheless, it is quite clear that even the hardest working and healthiest kidney can be overloaded by volume excess. Interstitial fluid shifting can result from generous substitution during surgery and is aggravated by increased vascular permeability [30]. In these cases, resulting edema is visible for surgeons and should be considered an alarm signal and threat to the patient, especially when risky anastomoses with known potential of fistulas need to be carried out. In the authors' opinion, this mechanism, which is supported by the findings presented in this study, seems intuitive and quite obvious. Consequently, anesthesiologists need to hand over part of the responsibility for adequate fluid management to surgeons and need to be aware that misguided fluid management can increase risk of surgical complications. The bottom line is that there is a good share of responsibility for surgical complications for the anesthesiologist and every discipline involved in the teamwork necessary to carry out complex surgical procedures needs to understand any intraoperative action contributes to success or failure.

Perioperative infusion management relies on both measurable parameters and experience. Fluid overload needs to be avoided, while fluids lost need to be replaced and hemodynamic parameters need to be manipulated to stabilize the patient during and after surgery [30]. During induction of anesthesia, a starting bolus volume is very often applied and is considered necessary to compensate for both hypovolemia of the fasting patient and vasodilatation during anesthesia or caused by epidural catheters [36–38]. This has been considered good practice for years but may be inappropriate and the foundation to postoperative complications even before surgery starts. During surgery, excessive volumes are infused to compensate loss to the third space, which has been an accepted concept for decades but may not even exist [39, 40]. Very often, sympathomimetic medication will be avoided since an impaired renal function is feared and crystalloids and colloids will be applied [41]. Intraoperatively, no significant difference was seen for the type of infusion solutions (i.e., crystalloids versus colloids) in our patients. Most interestingly, there was a striking difference in postoperative regimens: none of the patients with fistulas had received colloids during the first 72 h after surgery. Smaller volumes of colloids are needed to achieve the same effect compared to crystalloids; thus crystalloid substitution using colloids might be a worthwhile intervention, but data on this topic remains controversial [42]. It has to be remarked that in large meta-analyses no positive effect on survival and complications rates of colloids versus crystalloids could be identified; consequently, the usage of colloids has decreased over the last decade while the rate of pancreatic fistulas after PD with PJ remained unchanged [2, 43].

In a study from 2014, a threshold of 1 L positive fluid balance on postoperative day one was identified to be associated with an increased risk of complications after pancreatic surgery [28]. Since fluid balance during the first 24 h after surgery exceeded 1 L in any of our patients, this finding was not reproducible using our data.

## 5. Conclusion

Pancreatic surgery involves both sophisticated surgical and anesthesiological management, amongst other prerequisites [44–46]. Fluid management, which is within reach for interventions, needs to be considered as surgical and anesthesiological teamwork [30, 47]. Our analysis has demonstrated a clear association between intraoperative fluid excess and occurrence of pancreatic fistulas after PD with PJ even in patients with normal renal function. When in our patients the intraoperative infusion rate : GFR ratio exceeded 0.15, the rate of postoperative pancreatic fistulas was more than tripled. Our analysis points at an increased use of intra- and postoperative colloids as a possible intervention. High quality randomized clinical trials comparing different fluid regimens are needed to generate evidence in this important aspect of pancreatic surgery.

## Figures and Tables

**Table 1:** Patient characteristics.

	Postop. fistula (*n* = 18)		No postop. fistula (*n* = 65)		*p*
	*n*	%	*n*	%	
Sex					0.5956
Female	6	33	27	42	
Male	12	67	38	58	

	Median (IQR)		Median (IQR)		

Age [years]	65 (58.25–73)		66 (57–73)		0.8038
Creatinine [mg/dL]	0.9 (0.725–1.0)		0.8 (0.7–0.9)		**0.0074**
GFR [mL/min]	83 (61–98)		100 (79–115)		**0.0219**
Surgical duration [min]	231 (202–295)		230 (262–313)		0.5004
Intraoperative infusions					
Total [mL]	3250 (3000–4188)		3500 (3000–4000)		0.5626
Infusion rate [mL/min]	13.4 (10.7–17.0)		13.8 (10.8–16.1)		0.9634
INF : GFR	0.1688 (0.1397–0.2403)		0.1465 (0.1043–0.1743)		0.0585
Postop. infusions [mL/72 h]	12763 (11778.25–14718.75)		12500 (11548.75–14157.5)		0.8501

	*n*	%	*n*	%	

Usage of colloids					
Intraoperatively	10	56	28	43	0.4264
Postoperatively	0	0	20	31	**0.0046**

IQR: interquartile range; GFR: glomerular filtration rate; INF : GFR: infusion rate [mL/min] : GFR [mL/min] ratio.

**Table 2:** Rates of postoperative pancreatic fistulas for patients exceeding the infusion rate : GFR ratio.

	INF : GFR ≥ 0.15		INF : GFR < 0.15	
	*n* = 38	100%	*n* = 45	100%
Postoperative fistula	13	34	5	11
No postoperative fistula	25	66	40	89

INF : GFR: infusion rate [mL/min] : glomerular filtration rate [mL/min] ratio. *p* = 0.0157.
